# Escape Room Versus Traditional Simulation: A Comparative Study of the A-E (Airway, Breathing, Circulation, Disability, Exposure) Assessment in Undergraduate Medical Training

**DOI:** 10.7759/cureus.91934

**Published:** 2025-09-09

**Authors:** Naayema Hussaini, Umar Al-Haddad, Nabihah Hussaini, Ameenah Hussaini, Ihab Abdlaziz

**Affiliations:** 1 Internal Medicine, Guy's and St Thomas' NHS Foundation Trust, London, GBR; 2 Critical Care Medicine, Lewisham and Greenwich NHS Trust, London, GBR; 3 School of Medical Sciences, The University of Manchester, Manchester, GBR

**Keywords:** abcde approach, critically ill patients, escape room simulation, gamified simulation, medical education, simulation in medical education, traditional simulation

## Abstract

Background

The A-E (Airway, Breathing, Circulation, Disability, Exposure) assessment is a systematic approach used for the primary assessment of critically injured patients, and is often taught through simulation-based learning methods. The introduction of new educational methods, such as escape rooms, may be used to enhance engagement, teamwork, and educational value.

Objectives

The objective of this study is to compare the effectiveness of the conventional A-E simulation format with an escape room-style A-E simulation format in enhancing medical students’ engagement, teamwork, and educational value.

Methods

We conducted a qualitative study involving 19 undergraduate medical students, with nine allocated to the traditional simulation group and 10 to the escape room simulation group. The participants from the traditional simulation group and the escape room simulation group were further divided into three subgroups, with each group being assigned one of the following clinical scenarios: opioid overdose, sepsis, and upper gastrointestinal bleeding. Pre- and post-session questionnaires were used to assess engagement, teamwork, and educational value.

Results

Thematic analysis revealed four key themes: engagement and realism, teamwork and communication, clinical reasoning, and knowledge retention and enhanced learning. Escape room participants found the escape-style clinical scenarios to be more immersive and enjoyable in comparison to the traditional simulation group. The escape room-style simulation showed overall favourable reviews across the four key themes identified.

Conclusion

Escape room-style simulation is a highly engaging and interactive alternative to traditional A-E simulation in undergraduate medical education. It promotes learner engagement, teamwork, and clinical reasoning while maintaining educational rigour. Though more resource-intensive to develop, it holds promise as an effective adjunct for teaching A-E assessments.

## Introduction

The rapid assessment and management of critically ill patients is a core competency in medical education, particularly in acute care settings. One of the most widely adopted frameworks for structured patient assessment is the A-E (Airway, Breathing, Circulation, Disability, Exposure) approach [1]. This systematic approach to managing an acutely unwell patient is designed to help healthcare professionals identify and address life-threatening conditions in order of clinical urgency, ensuring that no critical aspect of the patient’s condition is overlooked. It is a unified language, understood by all healthcare professionals, and is applicable to any unwell patient regardless of underlying pathology [2].

Simulation-based teaching methods in medical education are widely used to practise A-E assessments, and studies have shown that this leads to improved patient outcomes [3]. To effectively train medical students in these high-stakes skills, simulation has become an essential component of modern medical curricula. Simulation provides a safe, controlled environment where learners can practise clinical reasoning, procedural skills, and teamwork without risk to real patients. It enhances knowledge retention, improves clinical confidence, and supports the development of non-technical skills, such as communication and leadership [4]. Traditional simulation often involves instructor-led scenarios using manikins or standardised patients, guided debriefings, and linear progression through clinical tasks [5].

In recent years, educators have begun to explore gamified simulation formats, such as medical escape rooms, as a novel way to engage learners. Inspired by recreational escape rooms, these educational experiences embed clinical challenges and puzzles within a time-limited, team-based format [6]. Learners must work collaboratively to interpret clues, make clinical decisions, and “solve” the case using their medical knowledge. Preliminary studies suggest that escape rooms can increase learner engagement, encourage teamwork, and improve clinical reasoning and acumen - all of which are critical in acute situations [7].

The objective of this study is to compare the effectiveness of the conventional A-E simulation format with an escape room-style A-E simulation format in enhancing medical students’ engagement, teamwork, and educational value.

## Materials and methods

Study design

This qualitative study explored undergraduate medical students’ experiences of two simulation formats: traditional simulation versus escape room-style simulation, using the A-E assessment approach. This study aimed to evaluate perceived engagement, teamwork, and educational value.

Medical students were randomly allocated to one of the two groups using a random number generator. Allocation was concealed from both participants and facilitators until just before the start of the simulation. Both simulations used identical clinical scenarios with consistent learning objectives, which differed only in delivery style. Pre- and post-simulation questionnaires (see Appendix 2) were used to assess engagement, teamwork, and educational value, using a Likert scale and open-ended questions, and analysed qualitatively. 

Participants and setting

A total of 19 undergraduate medical students were recruited voluntarily through an open invitation. Participants were randomly assigned to one of two groups: traditional simulation (n = 9) or escape room simulation (n = 10). Each group was further divided into three groups of three students (the opioid overdose escape room group had four students), with each group completing one clinical scenario: opioid overdose, sepsis, and upper gastrointestinal bleeding.

Simulation scenarios

Three emergency scenarios were used across both groups: opioid overdose, sepsis, and upper gastrointestinal bleeding. Scenarios were designed to assess the application of the A-E approach and were matched in clinical complexity, learning objectives, and duration across both simulation formats.

In the traditional simulation, students managed the scenario using a high-fidelity manikin, with guidance provided by a facilitator. The session was instructor-led and followed a linear A-E approach with real-time feedback. 

⁠In the escape room simulation, the same clinical cases were transformed into interactive challenges. Interactive challenges included puzzles, riddles, and locked boxes, all integrated into the scenario, and students had to identify clinical findings, perform key interventions, and work as a team to unlock equipment and progress through the case until they “escaped” the room, i.e., stabilised the patient. Appendix 1 shows the blueprint of the challenges we used.

In both simulations, instructors did not interfere with the flow of the simulation unless participants asked for help or if there was an obvious flaw in the A-E management that needed immediate intervention; otherwise, it was noted and discussed in the feedback at the end of the simulation. Prompts were posed as questions, such as “Is there anything else you would like to do in C (circulation)?”, to ensure standardisation and to allow for thinking time, in the hope that the students would figure out the answer themselves.

Data collection

Before and after the simulations, all participants completed pre- and post-simulation questionnaires. The pre-simulation questionnaire focused on previous simulation experience, confidence in conducting A-E assessments, and expectations. The post-simulation questions explored perceptions of engagement, teamwork, and the educational value of the simulation. These responses were used for data analysis. No identifying information was collected. The questionnaires included a mix of open-ended and Likert scale questions, allowing for qualitative analysis. The full questionnaires are available in Appendix 2. 

Data analysis

Responses to open-ended questions were analysed thematically using Braun and Clarke’s six-step approach to thematic analysis [8]. Initially, responses were read multiple times to familiarise ourselves with recurring phrases and keywords. Themes were developed directly from participants’ responses, allowing patterns to emerge from the data rather than from pre-existing categories. Themes were reviewed, grouped, and refined, and a final set of themes and subthemes was produced, capturing key patterns and insights across both simulation formats.

## Results

A total of 19 undergraduate medical students participated in the study, with nine students allocated to the traditional simulation group and 10 to the escape room simulation group. All participants completed the pre- and post-session questionnaires, providing qualitative data for analysis. Thematic analysis of open-ended student responses yielded four key themes: engagement and realism, teamwork and communication, clinical reasoning, and knowledge retention and perceived learning.

Engagement and realism

Students in the escape room group consistently described the experience as “fun,” “challenging,” and “memorable.” The gamified structure contributed to increased motivation and emotional engagement. In contrast, participants in the traditional simulation valued the realism and structured nature of the exercise, but reported a more subdued emotional experience. 

Teamwork and communication

Both groups recognised the importance of collaboration. In the escape room setting, communication strategies developed organically as students navigated tasks and self-assigned roles when it came to solving the puzzles. Teams were forced to self-organise, delegate roles, and adapt their approach to various scenarios in real time in order to progress through their tasks. The traditional simulation group relied more on facilitator prompts and feedback to structure their interactions, both with each other and with the simulated patient. 

Clinical reasoning

Escape room participants highlighted how the timed, puzzle-based format mimicked real-life pressure, requiring rapid interpretation of clinical cues and active problem-solving. Some described the puzzles as a “mental workout” that enhanced their decision-making skills. Conversely, participants in the traditional group appreciated the clear, step-by-step guidance, but reflected that the experience felt more passive and less cognitively demanding.

Knowledge retention and enhanced learning

Escape room participants reported improved recall of the A-E assessment, attributing this largely to the novelty and emotional intensity of the simulation format. The increased cognitive demand and need for self-organisation contributed to increased confidence in applying clinical knowledge under pressure in real-life medical scenarios. In contrast, students in the traditional group valued the structured debrief, and felt it reinforced clinical learning through guided reflection rather than active recall. 

Quantitative observations

While the qualitative focus of the study limits statistical comparison, Likert scale responses showed that escape room participants reported higher average ratings for engagement, teamwork, communication, working under pressure, problem-solving, confidence in managing A-E scenarios, and clinical reasoning. The Likert scale also showed that the escape room group would recommend the format to others in comparison to the traditional format. The traditional format was, however, more realistic in comparison to the escape room format. 

Likert scale responses provided a complementary perspective to the qualitative findings. Overall, participants of the escape room group reported consistently higher ratings across multiple domains of the simulation experience. Engagement was rated particularly high, with 93% of escape room participants selecting “agree” or “strongly agree” for the statement, “I found the simulation engaging,” compared to 67% in the traditional simulation group. Teamwork and communication were also rated more positively by the escape room participants, with 87% strongly agreeing that “the format supported effective teamwork and communication,” versus 77% in the traditional group.

When asked whether the session improved their confidence in managing acutely unwell patients, both groups showed a positive trend, but the increase was slightly more pronounced in the escape room cohort (escape room: 4.5/5 average rating and traditional: 4.1/5). Participants also rated their ability to work under pressure better in the escape room group compared to the traditional group (escape room group average 4.4/5 and 4.2/5 in the traditional group). The escape room group also showed a much higher score in problem-solving compared to the traditional group (escape room group average 4.6/5 and 3.9/5 in the traditional group).

The realism of the scenario was rated similarly in both formats (escape room: 4.2/5 and traditional: 4.4/5), suggesting that the gamified elements did not detract from the clinical credibility of the simulation. Clinical reasoning and decision-making were rated very similarly, with both traditional and escape room groups rating them 85% and 86%, respectively. Finally, overall satisfaction with the session and willingness to recommend the format to others was slightly higher in the escape room group, with 100% agreeing they would recommend it, compared to 87% in the traditional simulation group.

**Figure 1:**
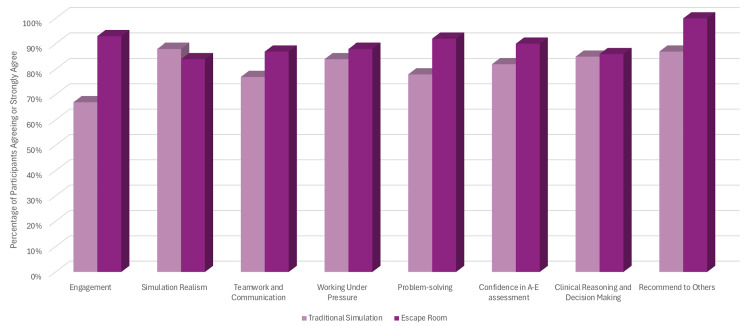
Comparison of Simulation Formats: Likert Scale Responses A-E: Airway, Breathing, Circulation, Disability, Exposure

These trends support the qualitative data, indicating that the escape room format was perceived as more engaging, teamwork-oriented, and conducive to applied learning without compromising on clinical value, as seen in Figure 1.

## Discussion

This study explored the impact of two simulation modalities, traditional simulation and escape room-style gamified simulation, on undergraduate medical students’ learning, engagement, teamwork, and clinical confidence in performing A-E assessments.

The findings suggest that, while both formats were perceived as valuable for clinical learning, engagement, and team building, the escape room format offered enhanced engagement, active participation, and cognitive involvement.

These observations align with growing literature supporting gamified learning in medical education. Escape rooms, as a form of alternative and novel teaching, have been shown to promote active participation, motivation, cognitive involvement, and improved knowledge retention, compared to more didactic methods [9-12].

Students from the escape room group highlighted the novelty, interactivity, and engagement of the format. Using puzzle-based scenarios in the A-E assessment allowed students not only to recall clinical knowledge but to apply it dynamically in a non-linear format, while simultaneously developing non-technical skills such as teamwork, communication, and decision-making. Unlike traditional medical teaching, escape room-style teaching allowed for creative thinking, adapting to uncertainty, and communicating effectively - all relevant in navigating the complexities of modern clinical practice [13,14]. This was evident during our simulations when students reported that the escape room format encouraged them to think creatively, engage with teammates, and collectively unpick complex clinical challenges. Students were also faced with making decisions related to time-pressured clinical challenges, many with incomplete information, that required prioritisation and applied clinical judgement, mirroring the dynamics of real-life clinical medicine.

In contrast, traditional simulation was described as more linear and facilitator-led. Although it is a familiar structure with valuable feedback, some students felt it lacked interactivity and novelty. Previous research similarly notes that, while traditional simulation enhances procedural and technical performance, it may be less effective in engaging learners when delivered rigidly [15].

However, the escape room style was not without challenges. A minority of students noted increased cognitive load due to ambiguous puzzle designs. This relates to the cognitive load theory, where unnecessary mental effort is directed toward decoding complex or irrelevant puzzles, rather than processing clinical information [16]. Overly complex puzzles can also lead to technical failures, equipment malfunction, or unexpected participant behaviour, which risks undermining the educational value of the simulation. To avoid this, we recommend piloting simulations beforehand, with clear contingency plans. Maintaining simplicity and clarity in puzzle design is key to ensure the integrity of the simulation and learning objectives.

Although gamification can enhance engagement and creativity, there is a risk that escape room simulations may become overly gamified, leading to confusion about the objectives. To mitigate this, escape room simulations must be aligned with clear learning objectives and supported by effective pre-briefing and debriefing, to consolidate learning and maintain focus [12]. Structured pre-briefing is crucial to emphasise that the goal is not simply to “escape,” but to apply clinical reasoning and teamwork effectively using the A-E approach. Clear debriefing reinforces key learning points and ensures the simulation is perceived as a serious clinical exercise, not just a game. In this study, structured debriefs were consistently cited as essential by both groups, reinforcing their role in bridging experience and reflection [9]. Each puzzle was designed to follow the A-E clinical assessment, with findings used to unlock clues, which was key in maintaining educational focus. Striking a balance between immersive gameplay and pedagogical clarity ensures that the educational value is not compromised by entertainment. This is supported by previous studies [17], which emphasise aligning escape room game design with educational goals to maximise value while maintaining engagement.

A small percentage of students found the escape room experience more anxiety-inducing in time-pressured situations, more so than in real life [18,19]. This further emphasises the need for simple puzzles, and clear pre-briefing and debriefing, reinforcing that the aim is clinical acumen, not game mechanics. Educators must ensure challenges reinforce learning objectives, rather than distract from them.

When comparing escape room-style simulation to traditional simulation, differences emerge in time and resource requirements. Traditional simulations are generally quicker to prepare, given their linear, facilitator-led format, and standardised debrief structure. Conversely, designing clinically accurate escape room simulations with meaningful puzzle-based challenges requires substantial time, creativity, and faculty input [9,20]. However, once developed, these materials can be reused with minimal adaptation, enhancing their long-term utility [21].

Just like other learner-centred approaches, such as the flipped classroom [22] and high-fidelity simulation [23], which are replacing traditional medical education models, the use of gamified simulation, particularly escape rooms, warrants further exploration. Although there is no current evidence that escape-room participants outperform others in summative A-E assessments, the greater engagement, teamwork, and cognitive challenge reported may translate into improved outcomes. Escape room-style simulations have already demonstrated educational value among nursing students and healthcare professionals [20].

Overall, this study contributes to the emerging evidence suggesting that escape room-style simulation can be a powerful adjunct to traditional methods in medical education, especially for developing non-technical skills such as teamwork, communication, applied clinical reasoning, and acumen.

## Conclusions

This study demonstrates that escape room-style simulation can be a highly engaging and effective method for teaching the A-E assessment to medical students. Compared to traditional simulation, the gamified format promoted greater enjoyment, teamwork, and cognitive engagement, with most participants reporting it as useful and stimulating. While no direct improvement in summative assessment outcomes was measured, the increased motivation and active participation observed suggest that this approach may enhance knowledge retention and clinical reasoning. As medical education increasingly embraces learner-centred and innovative teaching methods, integrating gamified simulation, such as escape rooms, offers a promising tool to complement traditional learning, particularly in simulation settings. Nonetheless, the effectiveness is comparable, with the findings applying only to short-term knowledge and skills gain, and further research with larger cohorts and objective performance metrics is needed to evaluate its long-term educational impact.

## Appendices

Appendix 1

Opioid Overdose

Scenario: A 24-year-old male was found unresponsive at home by his friends. Pupils pinpoint, respiratory rate (RR) 8, oxygen (O2) saturation of 89% on air.

Airway

Clinical issue: Decreased Glasgow Coma Scale (GCS) and snoring sounds lead to airway compromise.

Decreased GCS and snoring sounds prompt airway assessment. If students correctly perform a head tilt-chin lift or jaw thrust, they find a code on the manikin’s neck to unlock a box containing airway adjuncts.

Students need to correctly:

.      Recognise airway compromise. 

.      Perform adequate airway manoeuvres.

Breathing

Clinical issue: Respiratory depression with a RR of 8 and O2 saturation of 89% on room air.

Recognising the need to obtain RR and O2 saturations.

Once obtained correct RR of 8 and O2 saturations of 89%, there is a three-digit coded box. Inserting 889 opens the oxygen box, which contains a non-rebreather mask, a venturi mask and a nasal cannula. Students will then have to choose the most appropriate O2 mask therapy option, in this case it being a non-rebreather mask. 

Students need to correctly:

.      Obtain a respiratory rate.

.      Recognise to place an O2 probe and obtain O2 saturation.

.      Recognise that a respiratory rate of 8 is low, and a 89% is a low O2 saturation level. 

.      Select the non-rebreather mask as the most appropriate therapy for O2 delivery.

Circulation

Clinical issue: Blood pressure (BP) = 98/69, heart rate (HR) = 45, hypovolemia and bradycardia.

Recognising the need to obtain HR, BP and obtain vascular access. 

Once obtained correct HR of 45 and BP of 98/69, students are then expected to insert 459869 into a six-digit code found on a locked box. Inside the box, they’ll find an ECG machine, cannulas and blood bottles. Students are then expected to carry out an ECG, and mention that they would want to gain access and to take blood.

ECG will show sinus bradycardia. There is a locked box with the riddle: “I’m running slow, but I am in sync. What am I?” The answer is sinus bradycardia. Inserting sinus bradycardia in the answer box will give access to 250 mL, 500 mL, and 1 L 0.9% sodium chloride fluid bags. Students will have to select the most appropriate volume, in this case, a 500 mL fluid bag. 

Students need to correctly:

.      Obtain a heart rate. 

.      Recognise how to place a blood pressure cuff and obtain a blood pressure. 

.      Recognise that both the heart rate and blood pressure are low.

.      Carry out an ECG, by placing the leads correctly. 

.      Recognise the ECG shows sinus bradycardia. 

.      Selecting the most appropriate volume, the 500 mL fluid bag. 

Disability

Clinical issue: Reduced GCS.

Students are expected to accurately assess GCS (E2, V2, M4: total GCS = 8).

A locked box has a puzzle labelled “Unlock only if you know how to score the brain.”

They input the correct GCS of 8 into a lock, which unlocks the box. 

Inside the box, there is a drug chart for the patient, showing the patient having a high amount of opioids. 

Students are also expected to carry out a blood glucose check, but this has no effect on the gameplay. 

Students need to correctly:

.      Assess GCS. 

.      Check blood glucose levels.

Exposure

Clinical issue: Must complete a full examination of the patient.

When students expose the patient’s arms and legs (per standard E check), they find a code hidden on the back of the patient's right knee (ensuring they adequately exposed and assessed the patient). The code will open a box that contains naloxone, the antidote for opioid overdose. In applying the antidote, the patient starts waking up, and the main room door opens for students to “escape.”

Students need to correctly:

.      Expose and examine the patient fully, maintaining dignity. 

.      Know that naloxone is the antidote for opioid toxicity. 

.      Naloxone has a quick onset of action, and therefore, therapeutic effects will be seen quickly. 

Sepsis 

Scenario: An 86-year-old female patient was found confused at home with a two-day history of feeling unwell, feverish, and reduced urine output. Collateral history reveals the patient had dysuria a couple of days prior.

Airway

Clinical issue: The patient is talking but confused. Airway patent for now, but close monitoring needed. No challenge. 

Students need to correctly:

.      Know that if the patient is talking, the airway is patent. However, it will require close monitoring and repeated assessment.

Breathing 

Clinical issue: Respiratory physiological response to sepsis with a RR of 28 and O2 saturation of 91%.

Once obtained correct RR of 28 and O2 saturations of 91%, there is a four-digit coded box. Inserting 2891 opens the oxygen box, which contains a non-rebreather mask, a venturi mask and a nasal cannula. Students will then have to choose the most appropriate O2 mask therapy option, in this case it being a non-rebreather mask. 

Students need to correctly:

.      Obtain a respiratory rate.

.      Recognise to place an O2 probe and obtain O2 saturation.

.      Recognise that a respiratory rate of 28 is high, and a 91% is a low O2 saturation level. 

.      Select the non-rebreather mask as the most appropriate therapy for O2 delivery.

Circulation

Clinical Issue: BP = 86/52, HR = 112, capillary refill time (CRT) > 3s, signs of septic shock.

Recognise the need to obtain HR, BP, gain vascular access and take bloods, including a lactate and blood cultures.

Once obtained correct HR of 112 and BP of 86/52, students are then expected to insert 1128652 into a seven-digit code found on a locked box. Inside the box, they’ll find cannulas, blood bottles and blood culture bottles. Cannula sizes include 22G, 20G and 18G. Students must select the biggest cannula size, 18G, and mention they would like to obtain access, and take bloods, including a lactate and blood cultures. 

To obtain fluids, there is a locked intravenous (IV) fluid box, with the question: “Identify the source of infection.”

Nearby clues:

.      Urine dipstick (shows positive nitrites, leukocytes).

.      Foley catheter bag beside the bed with urine looking dark and filthy with sediments.

Inside the box, it gives access to 250 mL, 500 mL, and 1 L 0.9% sodium chloride fluid bags. Students will have to select the most appropriate volume, in this case, a 500 mL fluid bag. 

Students need to correctly:

.      Obtain a heart rate. 

.      Recognise how to place a blood pressure cuff and obtain a blood pressure. 

.      Recognise that the heart rate is high and the blood pressure is low. 

.      Recognise the need to take bloods, including a lactate and blood cultures. 

.      Recognise the need to obtain access, using the biggest cannula size. 

.      Selecting the most appropriate volume, the 500 mL fluid bag. 

.      Recognising that the urine dipstick is positive for nitrites and leukocytes points to a diagnosis of probable urinary tract infection (UTI).

Disability 

Clinical issue: Causes of delirium. 

Patient delirious, likely secondary to urosepsis. 

Although the blood glucose level will be normal, to ensure students don’t forget glucose, the puzzle will be based on glucose levels. Blood glucose levels will be 6.1.

A locked box has a puzzle labelled “the higher I am, the more sweeter I am”, in reference to blood glucose levels. 

They input the correct blood glucose level of 6.1 into a combination lock.

Inside the box, there is a copy of local antimicrobial guidelines. 

There is another locked box that contains the antibiotics, labelled “choose your fighter”. To unlock the box, the students need to have chosen the correct antibiotic from the guidelines. In this case, it was IV gentamicin. 

Students need to correctly:

.      Check blood glucose levels.

.      Correctly choose the appropriate antibiotic regimen from the guidelines.

Exposure 

Clinical issue: Must complete a full examination of the patient and identify signs of infection. 

A clue hidden on the patient’s skin and the temperature of the patient will reveal the code for the box. When students expose the patient’s arms and legs (per standard E check), they find a code hidden on the patient's back (ensuring they adequately exposed and assessed the patient). 

Students should also take the temperature of patient. The temperature of the patient is 40.1 degrees Celsius. The temperature will be the code to open the box. The code found on the patient's back is just a red herring to ensure students are adequately exposed and examine the patient. The code will open a box that contains a sheet that says “complete sepsis six to leave.”

When students write sepsis 6, the main room door opens for students to “escape.”

Students need to correctly:

.      Expose and examine the patient fully, maintaining dignity. 

.      Remember to take a temperature reading.

.      Know and apply sepsis 6.

Upper Gastrointestinal (GI) Bleed

Scenario: A 48-year-old man found dizzy and weak at home. History is not initially available. The patient has evidence of vomiting blood and melena.

Airway

Clinical Issue: Decreased GCS and blood in the mouth lead to airway compromise. 

Decreased GCS and "blood" fluid in the mouth prompt airway assessment. 

A clue card with a suction catheter is found near the manikin that reads:

“A river of red runs from the mouth-

Is it clear or is it clogged?

Protect the path or pay the cost,

For breath once lost, cannot be bought.”

Students must recognise that active vomiting of blood (haematemesis) requires airway protection due to aspiration risk. If students perform suctioning and perform airway manoeuvres, a code tucked inside the patient's mouth is revealed once the "blood" has been suctioned. 

The code will open a box that contains airway adjuncts for students to use. 

Students need to correctly: 

.      Recognise airway compromise. 

.      Suction the mouth. 

.      Perform adequate airway manoeuvres.

Breathing

Clinical Issue: Tachypnoea (RR of 24) due to hypovolemia. 

Students need to obtain the respiratory rate and O2 saturations. 

Once obtained correct RR of 24 and O2 saturations of 96%, on a nearby whiteboard, there is a formula: 

Code = (RR x 2) - 4. 

RR= 24. Therefore code is (24 x 2) - 4 = 44.

Code 44 unlocks the oxygen box, which contains a non-rebreather mask, a venturi mask and a nasal cannula. Students will then have to choose the most appropriate O2 mask therapy option, in this case it being a non-rebreather mask. 

Students need to correctly:

.      Obtain a respiratory rate.

.      Recognise to place an O2 probe and obtain O2 saturation. 

.      Recognise that a respiratory rate of 24 is high. 

.      Recognise that although 96% is within normal limits, the patient is acutely unwell and can deteriorate rapidly, and thus oxygen therapy is appropriate. 

.      Select the non-rebreather mask as the most appropriate therapy for O2 delivery.

Circulation

Clinical Issue: Hypovolemia (BP = 84/60) with signs of haemodynamic instability (HR = 133, CRT > 3s). 

Recognise the need to obtain HR, BP, gain access and take bloods, including a group and save. 

Once obtained correct HR of 133 and BP of 84/60, students are then expected to insert 1338460 into a seven-digit code. Inside the box, they’ll find cannulas and blood. Cannula sizes include 22G, 20G and 18G. Students must select the biggest cannula size, 18G, and mention they would like to obtain access, and take bloods, including a group and save. 

IV fluid box has a question: “What needs to be activated..”

In which the answer is major haemorrhage protocol, which opens a box containing 250 mL, 500 mL, and 1 L 0.9% sodium chloride fluid bags and packed red blood cell transfusion packs. Students will have to select the most appropriate volume, in this case, a 500 mL fluid bag and packed red blood cells. 

Students need to correctly:

.      Obtain a heart rate. 

.      Recognise how to place a blood pressure cuff and obtain a blood pressure. 

·      Recognise that the heart rate is high and the blood pressure is low.

·      Recognise the need to take bloods, including group and save. 

·      Recognise the need to obtain access, using the biggest cannula size. 

·      Activating major haemorrhage protocol. 

·      Selecting the most appropriate volume, the 500 mL fluid bag and packed red blood cells. 

Disability 

Clinical Issue: GCS drop due to hypovolemia

GCS = 14 (eg. E4 V4 M6)

A box is locked, labelled: “How awake is he?”

Students need to assess GCS and input “14” into a numerical lock, which will open the box. 

Inside the box, they’ll have local guidelines on how to manage upper GI bleeds. 

Students will have to do a blood glucose check, but this has no effect on the gameplay. 

Students need to correctly:

.      Assess GCS.

.      Check blood glucose levels.

Exposure 

Clinical Issue: Identify signs of blood loss and possible cause. 

Malena is hidden between the patient’s legs and underneath the bed sheets, so students have to take off the bed sheet and adequately expose and examine the patient.

There is a locked box labelled “what did you just find…” in which the answer is melena. 

The box contains the number for the gastro on-call team. 

When students call the gastro team, the main room door opens for students to “escape.”

Students need to correctly:

.      Expose and examine the patient fully, maintaining dignity. 

.      Recognise melena.  

.      Know the team to call for upper GI bleeds.

Appendix 2

Pre-simulation Questionnaire

Purpose: To assess participants’ baseline simulation experience, confidence with A-E assessment, and expectations for the simulation.

Demographics 

Year of study:

Have you previously participated in simulation-based teaching: Yes / No

If yes, approximate number of simulations: 

Have you ever participated in an educational escape room before?

If yes, approximate number of simulations:

Confidence and Familiarity

Please rate your confidence with the following using a scale from 1 (not confident at all) to 5 (very confident):

1. Performing an A-E assessment on an acutely unwell patient.

2. Recognising life-threatening conditions during assessment.

3. Responding to acute medical emergencies.

4. Working effectively in a clinical team under pressure.

5. Communicating effectively in emergency situations.

6. Applying clinical knowledge in emergency situations.

Expectations

1. What are your expectations for this simulation? 

2. What do you hope to learn or gain from this simulation?

Post-simulation Questionnaire 

Purpose: To assess participants’ perceptions of engagement, teamwork, and perceived educational value of the simulation format.

Which simulation format did you participate in? (Select one) 

.   Traditional Simulation

.   Escape Room Simulation

Perception of the Simulation 

Please rate the following statements on a scale from 1 (Strongly disagree) to 5 (Strongly agree):

1. I found the simulation engaging.

2. The scenario felt realistic and immersive. 

3. The simulation encouraged active participation.

4. The simulation supported effective teamwork and communication. 

5. The simulation improved my ability to make clinical decisions and work in a team under pressure.

6. The simulation encouraged active problem-solving. 

7. The simulation improved my confidence and understanding of how to manage an acutely unwell patient.

8. I was able to practise clinical reasoning and decision-making during the simulation. 

9. The debrief helped consolidate my learning.

10. I would recommend this type of simulation to other students.

Reflection and Feedback

1. What aspects of the simulation did you find most engaging or helpful?

2. What challenges did you face during the simulation? 

3. How did this simulation compare to other teaching formats you’ve experienced?

4. What would you improve about the simulation?

5. What key learning points will you take away from this experience?
